# Effects of Balance Training Combined With Either Transcranial Direct Current Stimulation or Stroboscopic Vision Training on Balance Performance in Individuals With Chronic Ankle Instability: A Systematic Review and Meta‐Analysis

**DOI:** 10.1002/jfa2.70210

**Published:** 2026-09-19

**Authors:** Ali Shamsi Majelan, Omid Shahani, Atekeh Keivani, Mahla Armanfar, Brice Picot

**Affiliations:** ^1^ Department of Sport Injuries and Corrective Exercise Faculty of Physical Education and Sports Sciences University of Guilan Rasht Iran; ^2^ SFMKS Laboratory Société Française des Masseurs Kinésithérapeutes du Sport Pierrefitte‐sur‐Seine France; ^3^ Inter‐University Laboratory of Human Movement Biology Savoie Mont Blanc University Chambéry France

**Keywords:** chronic ankle instability, function, postural control, vision

## Abstract

**Introduction:**

Despite the benefits of balance training, persistent postural deficits remain common in individuals with Chronic Ankle Instability (CAI). The aim of this study investigates the efficacy of Stroboscopic Vision Training (SVT) and transcranial Direct Current Stimulation (tDCS) as adjunct modalities to improve balance in this population.

**Methods:**

This review was developed in accordance with the PRISMA checklist. Five electronic databases (Web of Science, Scopus, PubMed, EBSCO, and Embase) along with three supporting search platforms and libraries (ScienceDirect, Cochrane Library, and Google Scholar) were searched from inception to June 20, 2026. Study quality was assessed using the PEDro scale, risk of bias with the RoB 2 tool, and the overall quality of evidence with GRADE. A meta‐analysis of the review was conducted using Comprehensive Meta‐Analysis v.3.0 software.

**Results:**

Ten studies including 353 participants were included in this review. The meta‐analytic results indicated that the effect sizes were 0.326 for static balance (tDCS = 0.303, SVT = 2.131) and 0.364 for dynamic balance (tDCS = 0.252, SVT = 1.086). The quality of the studies was high in the tDCS group and moderate in the SVT group. The risk of bias was higher in the SVT studies. The overall quality of the evidence was moderate in static balance and low in dynamic balance.

**Conclusions:**

Although both interventions show promise, the higher risk of bias in SVT studies suggests that tDCS is the preferred approach for rehabilitation when conditions are comparable. Nonetheless, clinical decisions should remain personalized.

**Trial Registration:**

PROSPERO with the number CRD420251260339

## 1 Introduction

Chronic ankle instability (CAI) is a condition characterized by persistent residual symptoms following an ankle sprain, such as loss of function, feelings of instability, and recurrences [1]. Epidemiological studies indicate that approximately 70% of individuals experience at least one ankle sprain during their lifetime, and 32%–74% of these individuals subsequently develop CAI [2, 3, 4]. According to the updated model proposed by Hertel and Corbett [1], CAI arises from the interaction of mechanical laxity, sensory‐motor dysfunction, and altered central processing, rather than ligamentous laxity alone. This model emphasizes that CAI leads to deficits in postural control, particularly during dynamic and dual‐task activities. If left inadequately treated, CAI can lead to recurrent ankle sprains, persistent sensations of joint instability or giving way chronic pain, muscle, reduced balance and postural control, and functional decline [5]. Although mechanical ankle instability refers to structural deficits, functional ankle instability (FAI) pertains to perceived instability despite a stable joint, whereas CAI is an inclusive clinical construct encompassing both mechanical and functional impairments [5].

Neurologically, injury to the mechanoreceptors of the lateral ankle ligaments disrupts proprioceptive input and impairs the efficiency of central nervous system (CNS) processing necessary for postural control and joint stability [2, 6]. Reduced sensory feedback compromises anticipatory muscle activation and limits effective motor responses during dynamic tasks [7]. Individuals with CAI demonstrate greater impairments in dynamic stability, postural control, and gait patterns during dual‐task activities, indicating deficits in cognitive–motor integration in addition to neuromuscular dysfunction [8]. Therefore, incorporating dual‐task conditions into assessment and rehabilitation may provide a more realistic evaluation of functional limitations and support the development of more comprehensive intervention strategies. Supporting this perspective, Simpson et al. [9] reported that CAI‐related impairments negatively affect neuromuscular control, resulting in diminished postural control and altered lower‐limb kinematics. Although balance training improves postural control in CAI, persistent deficits are often observed, and traditional assessments may fail to detect them [10, 11].

Postural stability relies on the integration of afferent input from the proprioceptive, visual, and vestibular systems [12]. The CNS adapts to changes in sensory availability through a process known as sensory reweighting, whereby greater emphasis is placed on the most reliable sensory cues while less reliable inputs are downregulated [13]. Although vision plays a crucial role in maintaining balance, excessive dependence on visual information may reduce postural control efficiency when visual input is compromised. Evidence indicates that individuals with CAI are more adversely affected by visual deprivation than healthy individuals, likely due to underlying somatosensory deficits [14]. Consequently, increased visual reliance may elevate the risk of injury during complex or visually demanding sports activities [15].

Stroboscopic Vision Training (SVT) has recently gained increasing attention in sports science and rehabilitation as a novel perceptual–motor training approach. Stroboscopic glasses, which intermittently alternate between transparent and opaque visual states, have been introduced as an emerging intervention to improve balance control in individuals with CAI [16, 17]. Balance training is known to reduce fall risk, whereas visual feedback plays a critical role in postural regulation. Modifying visual input during balance training enhances sensory challenges and promotes adaptive postural strategies [18]. Neurophysiological evidence suggests that SVT facilitates signal transmission along the dorsal visual stream, primarily via magnocellular pathways, enhances alpha‐phase reorganization in the occipital cortex, supports alpha–gamma cross‐frequency coupling, and increases predictive coding activity in the parietal cortex [19]. Six‐week stroboscopic balance training programs have been shown to increase cortical activity (theta and alpha bands), improve postural control, enhance proprioception, and improve functional stability and athletic performance in individuals with CAI [16, 20, 21]. Although static balance tasks may not adequately reveal visual dependence differences between CAI and healthy individuals, dynamic tasks consistently demonstrate greater visual reliance in CAI [22]. Compared with complete visual occlusion, stroboscopic vision appears to produce superior effects on sensory reweighting and functional stability [23].

Recent evidence from a double‐blind pilot randomized controlled trial reported that adding anodal transcranial direct current stimulation (tDCS) to a 4‐week balance training program did not produce clinically meaningful improvements in static postural control or self‐reported ankle function compared with sham stimulation, although small effects were observed for selected postural and neurophysiological measures [24]. When combined with balance training, tDCS may induce synergistic effects, although findings remain inconsistent and protocol‐dependent [25, 26, 27, 28].

The comparison of tDCS and SVT training is scientifically justified by their distinct, yet potentially complementary, effects on the neural processes underlying postural control. tDCS is designed to modulate cortical excitability and may influence the capacity of motor‐related networks to undergo task‐dependent plasticity during balance practice [29, 30]. In contrast, SV training imposes an intermittent visual‐input condition that increases the perceptual demands of movement and may promote the adaptive use of visual, proprioceptive, and vestibular information during postural regulation. Experimental evidence indicates that stroboscopic training can selectively improve visual motion sensitivity and transient attentional processing, although its effects are not uniform across all visual and attentional domains [31]. These different modes of action provide a scientific basis for examining whether balance training is enhanced more effectively by direct central neuromodulation or by a visually induced perceptual challenge. Such a comparison is clinically relevant because both interventions can be implemented as adjuncts to balance training, yet they may differ in their feasibility, therapeutic target, and capacity to produce transferable improvements in postural control. Consequently, this systematic review and meta‐analysis aim to assess the effectiveness of balance training paired with either SVT or tDCS in improving balance performance among individuals with CAI.

## 2 Materials and Methods

This study adhered to the PRISMA guidelines (Preferred Reporting Items for Systematic Reviews and Meta‐Analyses) [32] and followed the PRISMA‐in‐Exercise, Rehabilitation, Sport Medicine, and Sport Science (PERSiST) guidelines [33]. The Prospero registration number for this study is CRD420251260339. Compared with the original registered protocol, only the search time frame was updated and studies.

### 2.1 Search Strategy

Five electronic databases (Web of Science, Scopus, PubMed, EBSCO, and Embase) along with three supporting search platforms and libraries (ScienceDirect, Cochrane Library, and Google Scholar) were searched using the keywords. The search encompassed studies published in English from inception to June 20, 2026. This study aimed to comprehensively assess the impact of two distinct interventions on objective balance outcomes in individuals with CAI. The interventions included: (1) balance training paired with tDCS and balance training combined with SVT. The analysis utilized the PICOS framework, as follows:

P: Population: Individuals with CAI.

I: The intervention involves balance training that is enhanced through the application of either tDCS or SVT.

C: Comparison: Control groups receiving conventional exercise therapy alone, sham interventions, or no intervention.

O: Outcome: Balance ability (static and dynamic balance and postural control).

S: Study type: Randomized controlled trials (RCTs), and semi experimental.

Keywords were selected according to MeSH terms, and terms were drawn from published studies to complete the keyword set. The search keywords were categorized and reported in Table 1. In addition to the database search, a manual search was conducted to maximize the identification of relevant studies in this area. The comprehensive search strategy, including the full list of keywords and syntax applied across all databases, is detailed in Table S1.

**TABLE 1:** Search strategy.

Stage level	Keywords
#1	“Transcranial Direct Current Stimulation” OR “TDCs” OR “Electrical Stimulations” OR “Electroshock” OR “Non‐invasive brain stimulation” OR “Cathodal Stimulation” OR “Excitability”
#2	“Stroboscopic” OR “Strobe glasses” OR “Stroboscopic vision” OR “motion perception” OR “Motion tracking” OR “Visual skills”
#3	“Chronic ankle instability” OR “CAI” OR “Ankle sprain” OR “ankle injuries” OR “functional ankle instability” OR “Lateral ankle sprain”
#4	“Balance” OR “Postural control” OR “COP” OR “Dynamic” OR “Static” OR “Postural sway” OR “Stability” OR “Time to stability”
#5a	#1 AND #3 AND #4
#5b	#2 AND #3 AND #4

### 2.2 Eligibility Assessment

#### 2.2.1 Inclusion Criteria

Eligible studies involved participants with CAI as defined by the Intentional Ankle Consortium Statement (CAI individuals are defined as participants experiencing ongoing symptoms after an initial ankle sprain, such as LAS recurrences, episodes of giving way, or feelings of ankle joint instability) [5], studies were randomized controlled trials investigating either tDCS or SVT, both combined with balance training, in individuals with CAI and with outcomes including both static and dynamic balance measures across various tests or instruments. Only articles written in English were included and the control group had to consist of participants receiving no exercise, exercise therapy alone, or exercise therapy plus a sham intervention.

#### 2.2.2 Exclusion Criteria

Exclusion criteria comprised: (1) studies lacking a non‐invasive brain stimulation or visual training intervention, (2) non‐peer‐reviewed gray literature including conference proceedings, thesis dissertations, trial protocols, and preprints lacking complete methodological reporting or peer‐reviewed status, and (3) systematic reviews, narrative reviews, and non‐English publications.

### 2.3 Study Selection and Data Extraction

After searching the databases, all results were entered into EndNote version 20 [34]; duplicates were identified and removed. In accordance with inclusion and exclusion criteria, titles and abstracts were screened by two authors (O.S. and A.S.M.). Following this step, the remaining studies were assessed in full text for eligibility. Disagreements were resolved by a third author (A.K.), and consensus was reached among the three authors; the final report reflects the consensus of all three authors relevant to this section. Author, year of publication, and country where the study was conducted; study objectives; participant characteristics (sample size, sex distribution, and mean age); intervention characteristics (type of intervention, duration, session frequency, session length, stimulation parameters, and exercise protocol); comparison group characteristics; balance assessment methods and other outcome measures; primary outcome variables; and the main findings of each study were extracted and recorded. For studies including multiple intervention or control arms, data were extracted separately for each group to preserve methodological consistency. The mean, standard deviation, and sample size for each group were also extracted to calculate effect sizes for the meta‐analysis. Two authors (O.S. and A.K.) participated in the data extraction process, and any disagreements were reviewed by a third author (M.A.F.); disagreements were resolved by consensus among the authors, and the reports were recorded. Studies that did not provide clear data were requested by email and included in the study if the information was received. For studies presenting data graphically, data extraction was performed using GetData software (V2.26) [25].

### 2.4 Quality Assessment

The methodological quality of the included studies was evaluated using the PEDro scale. Scores of seven and above were classified as excellent, scores between five and six as moderate, and scores of four and below as poor. The scale ranges from 0 to 10 [35]. As per standard PEDro scoring protocol, Item 1 (eligibility criteria) relates to external validity and was not included in the calculation of the final total score (maximum score of 10). Each study was independently assessed by two authors (O.S. and A.K.), with any disagreements resolved by a third author (M.A.F.), and the final report was documented by consensus among the authors.

### 2.5 Bias Risk Assessment

The Cochrane Risk of Bias Tool, Version 2 (RoB 2), was used to assess the risk of bias in the included studies. This tool evaluates five domains and provides a final judgment in three categories: “low,” “some concerns,” or “high” [36]. Two reviewers (O.S. and A.K.) independently assessed each study, and in the event of disagreement regarding the score or the study itself, a third reviewer (M.A.F.) re‐evaluated the study to reach a consensus among the authors then final assessment was recorded.

### 2.6 Certainty of Evidence

The certainty of evidence for each section was appraised using the GRADE (Grading of Recommendations, Assessment, Development, and Evaluation) framework. For each primary outcome, the evaluation incorporated five criteria: risk of bias, inconsistency, indirectness, imprecision, and publication bias [37].

### 2.7 Statistical Analysis

Statistical analyses were performed using Comprehensive Meta‐Analysis (CMA) version 3.7. To ensure methodological rigor and minimize confounding, outcomes were categorized into two distinct groups based on their measurement characteristics: (1) static balance and (2) dynamic balance. For each outcome category, a meta‐analysis was conducted to evaluate the overall effect of interventions. To investigate potential differences between intervention types, a subgroup analysis was performed, categorizing studies into tDCS and stroboscopic vision training. The effect size was calculated using Hedges' *g* to account for potential small sample size bias (< 0.20), small (0.20–0.49), moderate (0.50–0.79), and large (≥ 0.80) [25]. For each analysis, statistical heterogeneity was assessed using the *I* ^2^ statistic and the Cochran's *Q* test (0%–40% might not be important, 30%–60% may represent moderate heterogeneity, 50%–90% may represent substantial heterogeneity, and 75%–100% considerable heterogeneity) [38]. Given the anticipated clinical and methodological heterogeneity arising from variations in stimulus parameters, exercise intensities, and population demographics inherent in sports science research a random‐effects model was strictly applied. Unlike the fixed‐effects model, the random‐effects approach was deemed more appropriate to provide a more conservative and generalizable estimate of the intervention effect, accounting for the distribution of true effects across the diverse study settings [25]. Sensitivity analyses were conducted to assess the impact of inter‐outcome correlation on the pooled estimates; although the primary analysis assumed a correlation coefficient of *r* = 0.5, sensitivity scenarios were tested using *r* = 0.25 and 0.75. Due to the limited number of studies included in each subgroup and the overall analysis, formal assessment of publication bias via funnel plots and statistical tests Begg's test was deemed not feasible and was therefore not performed, as these methods lack sufficient power in small‐sample meta‐analyses. All statistical significance was set at < 0.05.

## 3 Result

### 3.1 Search Results

**FIGURE 1:**
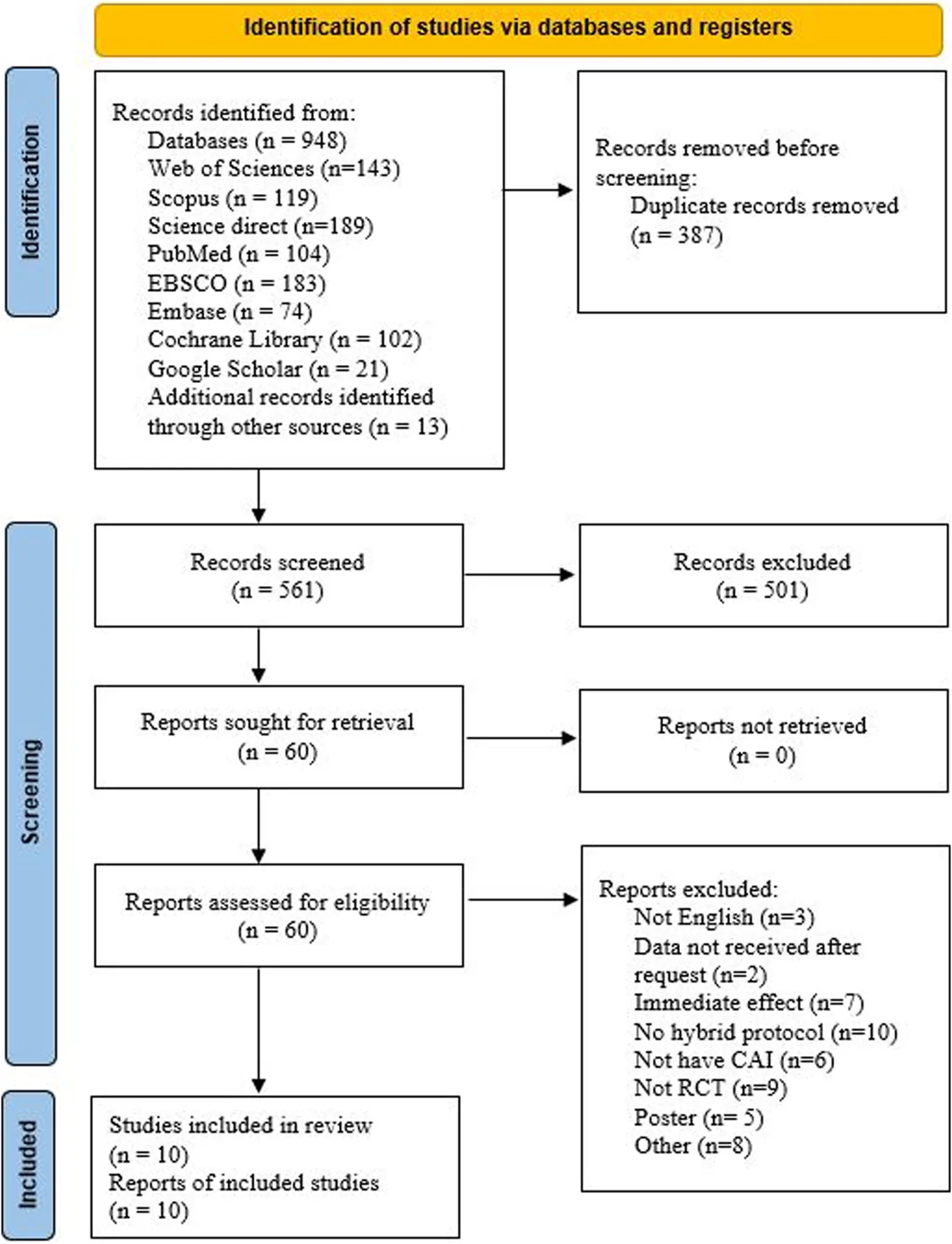
Flow diagram.

The initial search procedure identified 948 studies. After removing duplicate or similar records (*n* = 387), 561 studies progressed to the next stage. In the following stage, the titles and abstracts were screened by two authors (O.S. and A.S.M.), resulting in the exclusion of 501 studies (consistency rate = 98.23%, *κ* = 0.89). Ultimately, 60 studies were included for full‐text review. In accordance with the predefined inclusion and exclusion criteria, two authors reviewed the full texts of the remaining studies (O.S. and A.S.M.), and 10 studies met the inclusion criteria (consistency rate = 93.18%, *κ* = 0.81), 5 focusing on SVT, and 5 on tDCS. Figure 1 provides a visual representation of the study selection process. The reasons for excluding studies are described in the flow‐chart diagram. Additional information is provided in Figure 1. Additional information for each study is reported in accordance with the items listed in Table 2.

**TABLE 2:** Summary of studies conducted on the effect of tDCS and STV on balance ability individuals with CAI.

Article		Sample			Intervention				
Author(s) (year/country)	Objective	Number (M/F)	Age (y) mean ± SD	Duration (weeks/session per week	Type of intervention	Stimulation parameters	Type of balance and other assessment	Outcome	Result
Beyraghi and Khanmohammadi (Iran/2025) [26]	Investigate the effects of concurrent BT and tDCS over the supplementary motor area on anticipatory postural adjustments during gait initiation in persons with CAI	Experimental group, *n* = 16 (12/4)	27.59 ± 6.45	12 sessions; 4 weeks; 3/week; 60 min/session	BT + real tDCS over SMA (single limb stance, limb stance with kicking ball, single limb hop to stabilization, hop to stabilization and reach)	1.5 mA, 20 min, 12 sessions, SMA	Force platform analysis during gait initiation	COP displacement and velocity	Both groups showed improvements in gait initiation parameters; tDCS provided no significant additional benefit
		CG, *n* = 16 (11/5)	27.46 ± 5.54	12 sessions; 4 weeks; 3/week; 60 min/session	BT + sham tDCS	—			
Kim et al. (2026/USA and South Korea) [24]	Evaluate whether anodal tDCS has additive effects on static postural control, neural excitability, and perceived ankle function in individuals with CAI compared with sham stimulation during BT	Experimental group, *n* = 9 (5/4)	23.11 ± 8.19	12 sessions; 4 weeks; 3/week; 20 min/session	BT + real anodal tDCS (2 mA) over contralateral motor cortex (Cz) (progressive barefoot BT: single‐leg stance, single‐leg Romanian deadlift, and wobble board exercise)	2 mA, 20 min, 12 sessions, M1 (contralateral)	Static balance: Center of pressure (COP) velocity and area (eyes open/closed) Neural excitability: MEP of Soleus (SL), Fibularis Longus (FL), Tibialis Anterior (TA), and H/M ratio Perceived function: FAAM‐ADL and FAAM‐sport	COP displacement and velocity, corticospinal excitability, spinal excitability, perceived ankle function	COP velocity, COP area, MEP of SL, and FAAM scores Both groups improved over time. Active tDCS demonstrated only small additional effects; no clear additive benefit compared with sham stimulation
		CG, *n* = 6 (2/4)	22.33 ± 6.74	12 sessions; 4 weeks; 3/week; 20 min/session	BT + sham tDCS (30‐s ramp‐up/down) (identical progressive barefoot BT)	—			
Beyraghi et al. (Iran/2025) [27]	Explore whether adding tDCS to BT enhances preparatory brain activity and clinical outcomes in CAI	Experimental group, *n* = 15 (11/4)	27.47 ± 6.57	12 sessions; 4 weeks; 3/week; 60 min/session	BT + real tDCS (single limb stance, limb stance with kicking ball, single limb hop to stabilization, hop to stabilization and reach)	1.5 mA, 20 min, 12 sessions, SMA	EEG (CNV), SEBT, APA duration, clinical scales	Late CNV amplitude, CNV peak amplitude, CNV peak time, APA duration, dynamic balance, severity of perceived ankle instability	BT improved CNV, dynamic balance, and instability perception. tDCS showed no additional effect
		Control group, *n* = 15 (10/5)	28.41 ± 5.58	No training	BT + sham tDCS	—			
Ge et al., (China/2025) [28]	Investigate effects of HD‐tDCS combined with Bosu ball training on static and dynamic postural stability in CAI	Experimental group, *n* = 18 (M)	20.1 ± 1.3	3×/week, 20 min/session, 6 weeks (18 sessions)	HD‐tDCS + BBT (single‐leg stance, single‐leg stance with swing forward‐backward, single‐leg stance with swing medial‐lateral, swallow balanced stance, single‐legged squat and take‐ups, catching a ball while single‐leg stance, bending over to touch the edge while single‐leg stance)	2 mA, 20 min, 18 sessions, HD‐tDCS over Cz (M1/S1)	Force platform (single‐leg stance, drop landing)	CoP_RMS (static), TTS (dynamic)	Both groups improved static and dynamic stability; HD‐tDCS + Bosu group showed greater reductions in ML CoP_RMS and TTS compared with Bosu alone
		CG, *n* = 16 (M)	21.0 ± 1.8	3×/week, 20 min/session, 6 weeks (18 sessions)	Sham HD‐tDCS + BBT	—			
Zhang et al., (China/2025) [39]	Compare tDCS, BT, and combined effects on static and dynamic postural control in athletes with CAI	Experimental Group 1, *n* = 9 (5/4)	20.56 ± 1.67	12 sessions over 4 weeks (3/week)	tDCS	2 mA, 20 min, 12 sessions, Cz (anode)/Fp2 (cathode)	BESS, YBT	BESS total, YBT composite reach (COMP), error scores (Sfi, Sfo, Tfi, Tfo), YBT reach distances (ANT, M, PL)	Static balance improved in all intervention groups; dynamic balance improved only in groups with BT; tDCS showed no added benefit
		Experimental Group 2, *n* = 9 (5/4)	20.00 ± 1.00	12 supervised sessions over 4 weeks (3/week)	tDCS + BT (single‐limb hops to stabilization, hop to stabilization and reach, unanticipated hop to stabilization, single‐limb stance eyes open, single‐limb stance eyes closed)				
		CG 1, *n* = 9 (5/4)	20.78 ± 1.48	12 supervised sessions over 4 weeks (3/week)	Sham tDCS	—			
		CG 2, *n* = 9 (5/4)	19.78 ± 0.97	12 supervised sessions over 4 weeks (3/week)	Sham tDCS + BT	—			
Kim et al., (Korea/2021) [23]	Evaluate the effectiveness of partial visual deprivation training in individuals with CAI	Experimental Group 1, *n* = 25 (12/13)	29.76 ± 10.009	18 sessions over 6 weeks (3/week) (20 min)	BT (crossed arms and single‐limb stance, throwing/catching a ball. Single‐limb on the floor, single leg deadlift with open arms, lateral hop to stabilization hands at the hip, back and forward hop to stabilization with hands at the hip, randomized hop to stabilization in four directions)	—	SEBT, ankle dorsiflexion range of motion, self‐reported instability feeling, ankle functional status	SEBT (Ant/PM/PL), DFROM, CAIT, FAAM	SV and BT improved outcomes compared with control; SV training showed added benefit for instability and dynamic balance, suggesting potential value in CAI rehabilitation
		Experimental Group 2, *n* = 24 (17/7)	27.38 ± 7.383	18 sessions over 6 weeks (3/week) (20 min).	BT + SV	Stroboscopic glasses (senaptec), progressive level 1–8, 18 sessions, 20 min/session			
		CG, *n* = 24 (13/11)	29.67 ± 9.407	No training	No training	—			
Lee et al., (USA/2021) [40]	To compare the effects of BT with and without SV on postural control, and to examine the impact of such training on visual reliance in individuals with CAI	Experimental group, *n* = 14 (6/8)	21 ± 3	12 supervised sessions over 4 weeks (3/week) (20 min)	BT + SV (single‐limb hops to stabilization, hop to stabilization and reach, unanticipated hop to stabilization, single‐limb stance EO, single‐limb stance EC)	SVT (senaptec), 3 Hz (0.1 s clear/0.233 s opaque) during testing; 12 sessions	Force plate, YBT, Romberg analysis	Static postural control; DPSI (single‐leg hop); YBT; visual conditions (EO, EC, SV); Romberg ratios (SV/EO, EC/EO)	SV group improved postural control indices and reduced visual reliance; significant gains in ML velocity and YBT‐anterior
		CG, *n* = 14 (8/6)	22 ± 2	12 supervised sessions over 4 weeks (3/week) (20 min)	BT	—			
Uzlaşır et al., (Turkey/2021) [41]	Examine cortical activity and balance velocity after SV training	Experimental Group 1, *n* = 13 (7/6)	19.08 ± 0.40	3 days/week for 6 weeks (15–20 min/session)	BT + SV (single‐leg hop to stabilization, hop to stabilization and reach, unanticipated hop to stabilization, single‐leg balance)	SVT (senaptec), level 3 (100/150 ms), 18 sessions	EEG (21‐channel), HUBER balance device (COP‐v during stance transition)	Cortical activity, balance velocity	SV + BT led to increases in cortical activity (Cz theta and alpha) and center of pressure velocity, showing greater improvements compared with non‐SV and control groups
		Experimental Group 2, *n* = 13 (7/6)	20.46 ± 0.51	3 days/week for 6 weeks (15–20 min/session)	BT	—			
		CG, *n* = 13 (6/7)	20.23 ± 0.39	No training	No training	—			
Uzlaşır et al., (Turkey/2024) [20]	Effects of 6‐week SV + BT on balance in athletes with CAI	Experimental group, *n* = 13 (7/6)	19.08 ± 1.44	3 days/week for 6 weeks (15–20 min/session)	BT + SV (single‐limb hop to stabilization, hop to stabilization and reach, unanticipated hop to stabilization, single‐limb stance)	SVT, level 3 (100/150 ms), 18 sessions	HUBER device (single‐leg stance, LOS test)	COP length/area, LOS	SV training significantly improved dynamic balance (LOS); no improvement observed in static balance
		CG, *n* = 13 (7/6)	20.46 ± 1.85	3 days/week for 6 weeks (15–20 min/session)	BT	—			
Choi et al., (Korea/2024) [16]	Investigate effects of SV added to performance functional training	Experimental group, *n* = 20 (9/11)	23.35 ± 3.96	3 days/week for 8 weeks (30 min/session)	Proprioceptive feedback training + SV (single‐limb jumps to stabilization, jump to stabilization and reach, unexpected jump to stabilization, single leg balance)	SVT (senaptec), level 3 (100/150 ms), 24 sessions	COP‐v (mm/s, velocity balance test), JPS errors (inversion, eversion, dorsiflexion, plantarflexion)	COP‐v	PFT + SG group showed superior improvements in FAAM‐S, IdFAI, JPS, BVT, and FPTs compared with PFT group
		CG, *n* = 20 (10/10)	24.85 ± 3.65	3 days/week for 8 weeks (30 min/session)	Proprioceptive feedback training	—			

Abbreviations: ANT, anterior; APA, anticipatory postural adjustment; BBT, Bosu ball training; BESS, Balance Error Scoring System; BT, balance training; BVT, balance velocity test; CAI, chronic ankle instability; CAIT, Cumberland Ankle Instability Tool; CG, control group; CNV, contingent negative variation; COMP, composite reach; COP, center of pressure; COP RMS, root mean square of center of pressure displacement; COP‐v, center of pressure velocity; DFROM, dorsiflexion range of motion; DPSI, Dynamic Postural Stability Index; EC, eyes closed; EO, eyes open; FAAM, foot and ankle ability measure; FAAM‐ADL, foot and ankle ability measure‐activities of daily living; FAAM‐S, foot and ankle ability measure‐sports; FPT, functional performance tests; GRF, ground reaction force; HD tDCS, high definition transcranial direct current stimulation; IdFAI, identification of functional ankle instability; JPS, joint position sense; LOS, limits of stability; M1, primary motor cortex; ML, mediolateral; PHSB, proprioceptive and high speed balance training; PL, posterolateral; PM, posteromedial; SEBT, star excursion balance test; Sfi, single foot inside; Sfo, single foot outside; SMA, supplementary motor area; SV, stroboscopic vision; SVT, stroboscopic vision training; Tfi, two foot inside; Tfo, two foot outside; TTS, time to stabilization; YBT, Y balance test.

### 3.2 Study Characteristics

The 10 included studies were published between 2021 and 2026 with a total number of 353 participants (214 males and 139 females). The mean age range of participants in these studies was from 17.5 to 40 years. Of the five tDCS studies, 4 conducted a training protocol over 4 weeks and 1 over 6 weeks. Of the five SVT studies, 1 was conducted over 4 weeks, 3 over 6 weeks, and 1 over 8 weeks.

### 3.3 Quality Assessment

The quality assessment of the studies was conducted using the PEDro scale. The average score across included studies was 7.1 out of 10 on the PEDro scale, indicating relatively high methodological quality. The mean score for the tDCS studies was 7.2 and the SVT studies was 6.4 out of 10. Among the studies, two were of lower quality (scored 5) [15, 16], and two were of higher quality (scored 9) [20, 21]. Further details of the quality assessment are reported in Table 3.

**TABLE 3:** PEDro scale.

Study	1	2	3	4	5	6	7	8	9	10	11	Total (/10)
Transcranial direct current stimulation												
Beyraghi and Khanmohammadi [26]	1	1	1	1	1	0	0	1	0	1	1	7
Ge et al. [28]	1	1	0	1	1	0	0	1	0	1	1	6
Zhang et al. [39]	1	1	0	1	1	0	1	1	0	1	1	7
Beyraghi et al. [27]	1	1	1	1	1	0	0	1	1	1	1	8
Kim et al. [24]	1	1	1	1	1	1	0	0	1	1	1	8
Stroboscopic vision training												
Uzlasir et al. [41]	1	1	1	1	1	0	1	1	1	1	1	9
Uzlasir et al. [20]	1	1	1	1	1	0	1	1	1	1	1	9
Choi et al. [16]	1	1	0	1	0	0	1	0	0	1	1	5
Kim et al. [23]	1	1	1	1	0	0	1	1	0	1	1	7
Lee et al. [40]	1	1	0	1	0	0	0	1	0	1	1	5

*Note:* (1) Eligibility criteria, (2) random allocation, (3) concealed allocation, (4) groups similar at baseline, (5) participant blinding, (6) therapist blinding, (7) assessor blinding, (8) < 15% dropouts, (9) intention‐to‐treat analysis, (10) between‐group difference reported, and (11) point estimate and variability reported. 1 = yes, 0 = no.

### 3.4 Risk of Bias

**FIGURE 2:**
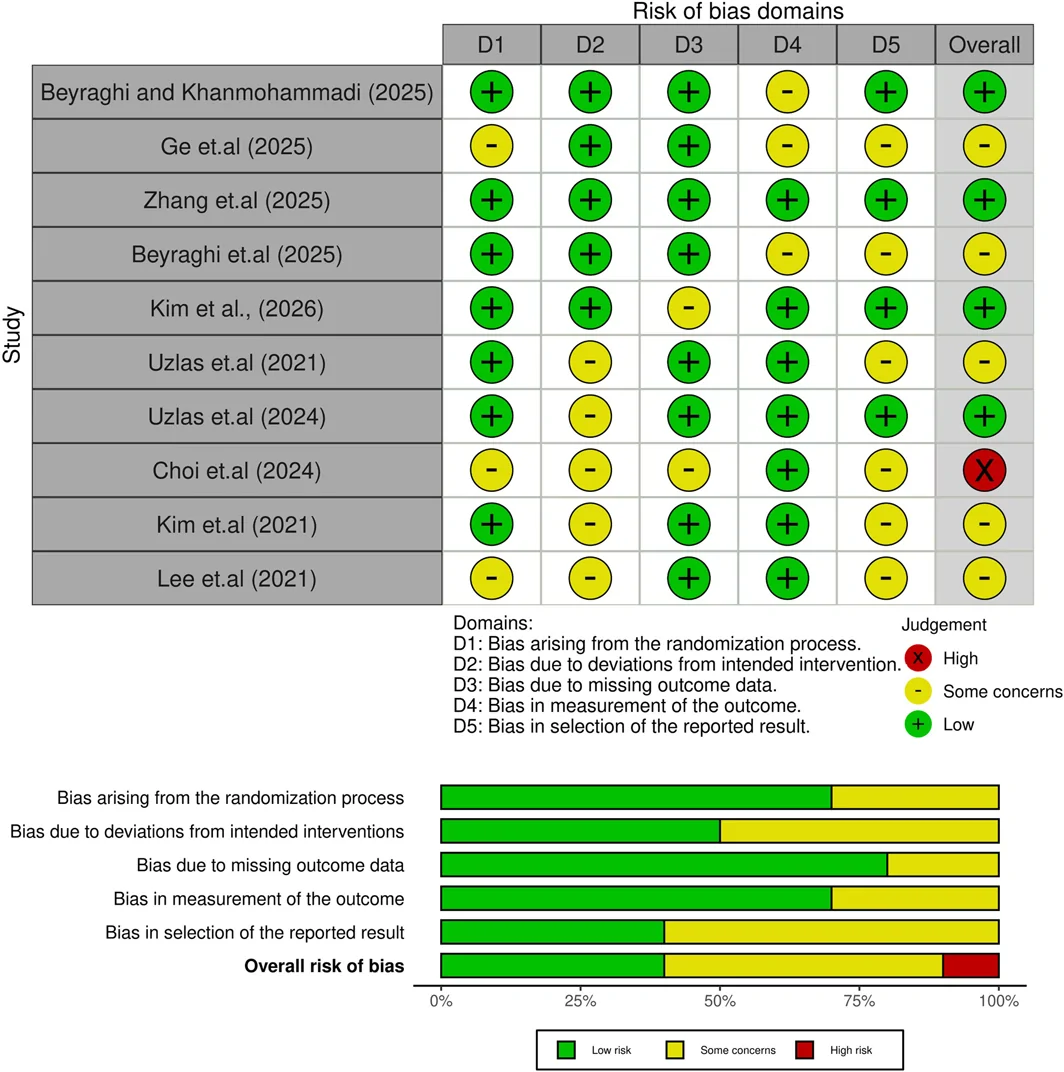
Analysis of risk of bias.

Study bias is reported in Figure 2. Each study was reviewed separately, and only one study exhibited a high level of bias [16]. The studies in the tDCS group were more favorable than those in the SVT group, with two studies in the tDCS group having some concerns [27, 28] and three studies having a low level of bias [24, 26, 39]. In the SVT group, one study had a low bias [20], three studies had some concerns [15, 21, 23], and one study had a high bias [16]. Overall, 40% of the studies had a low level of bias, 50% had some concerns, and 10% had a high level of bias.

### 3.5 Narrative Description of Included Interventions

Overall, the qualitative synthesis of extracted data (Table 2) revealed distinct trend patterns between the two adjunct interventions. For tDCS combined with balance training, the majority of studies employing conventional montages [24, 26, 27, 39] reported no significant additive benefits over balance training alone or sham tDCS. Conversely, targeted high‐definition tDCS (HD‐tDCS) demonstrated statistically significant improvements in postural stability [28]. In contrast, SVT consistently demonstrated positive additive effects across all reviewed studies [16, 20, 21, 23, 40], showing uniform enhancements in dynamic balance, neuroplastic adaptations, and patient‐reported ankle function.

### 3.6 Data Synthesis

#### 3.6.1 Static Balance

**FIGURE 3:**
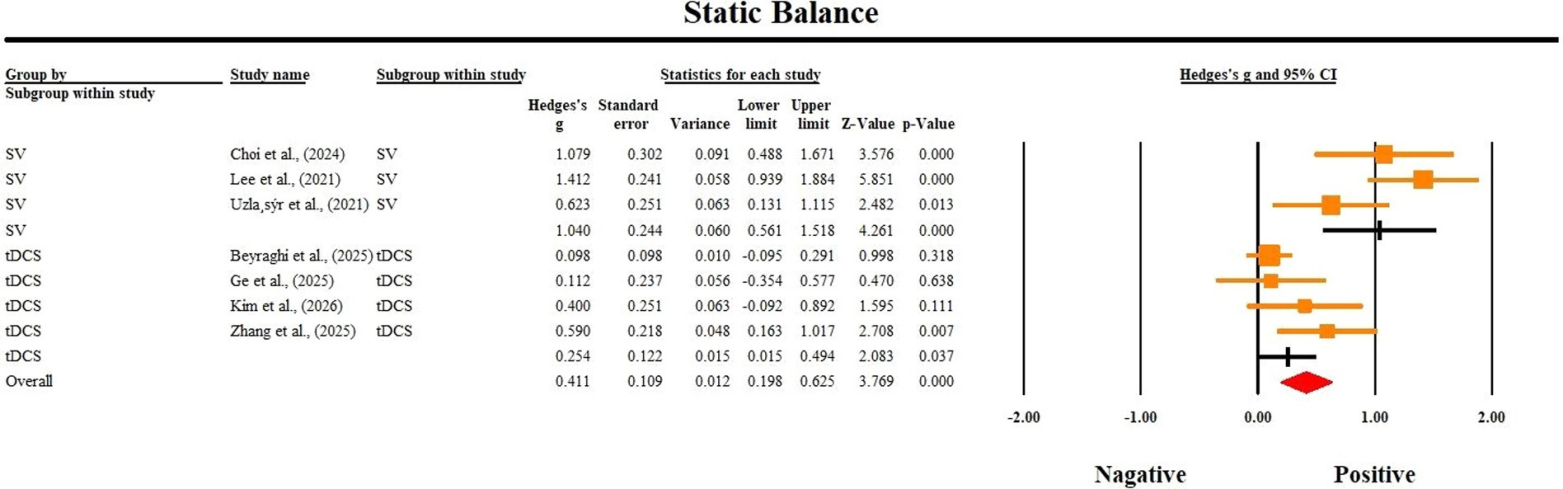
Static balance analysis.

A meta‐analysis was conducted to evaluate the overall effect of tDCS and SV training on static balance. Seven studies met the inclusion criteria. Utilizing a random‐effects model necessitated by significant heterogeneity (*I* ^2^ = 86.95%, *p* = 0.0001) the pooled effect size was 0.326 (95% CI: 0.167, 0.485; *Z* = 4.028; *p* = 0.0001). Subgroup analysis was performed to explore differences between intervention types. The tDCS subgroup (*n* = 4) yielded an effect size of 0.303 (95% CI: 0.143, 0.463; *p* = 0.0001), showing no significant heterogeneity (*I* ^2^ = 0.0%, *p* = 0.745). In contrast, the SVT subgroup (*n* = 3) showed a larger effect size of 2.131 (95% CI: 0.718, 3.544; *p* = 0.003), with substantial heterogeneity (*I* ^2^ = 88.06%, *p* = 0.0001). This observed heterogeneity may stem from variations in intervention duration, gender distribution, and administration frequency. Due to the limited sample size (*n* = 7), a formal assessment of publication bias via funnel plots or statistical tests was not feasible. Detailed results are presented in Figure 3.

#### 3.6.2 Dynamic Balance

**FIGURE 4:**
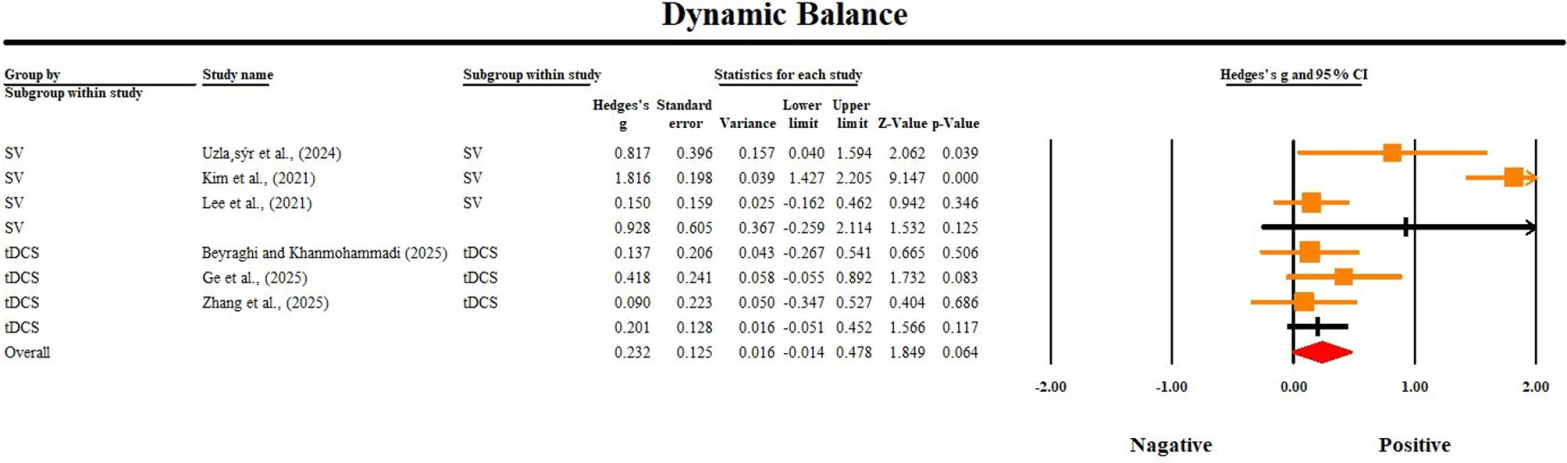
Dynamic balance analysis.

A meta‐analysis was conducted to evaluate the overall effect of tDCS and SV training on dynamic balance. Six studies were included in the analysis. Utilizing a random‐effects model as necessitated by significant heterogeneity (*I* ^2^ = 79.15%, *p* = 0.0001) the pooled effect size was 0.364 (95% CI: 0.031, 0.697; *Z* = 2.144; *p* = 0.032). Subgroup analysis was performed to compare the two intervention types. The tDCS subgroup (*n* = 3) yielded an effect size of 0.252 (95% CI: −0.106, 0.610; *p* = 0.168), with no significant heterogeneity observed (*I* ^2^ = 0.0%, *p* = 0.713). Conversely, the SVT training subgroup (*n* = 3) showed an effect size of 1.086 (95% CI: 0.179, 1.993; *p* = 0.019), characterized by substantial heterogeneity (*I* ^2^ = 84.69%, *p* = 0.001). The observed heterogeneity in the SVT group may be attributed to variations in intervention protocols specifically duration and session frequency as well as discrepancies in the types of glasses utilized. Due to the limited number of studies (*n* = 6), a formal assessment of publication bias via funnel plots or statistical tests was not feasible. Detailed results are presented in Figure 4.

#### 3.6.3 Sensitivity Analysis

To evaluate the potential impact of dependency between outcomes on our results, a sensitivity analysis was conducted by adjusting the assumed correlation coefficient (*r*) from 0.25 to 0.75. The stability of the synthesized effect sizes across these scenarios confirms that the observed outcomes are not unduly influenced by the assumed level of inter‐outcome correlation. This demonstrates the robustness of the meta‐analytic estimates. Detailed results of the sensitivity analyses are provided in Supporting Information S1.

### 3.7 Certainty of Evidence

The certainty of balance ability was assessed across two section of studies. In the static balance, certainty was moderate. In the dynamic balance, certainty was low. Details are reported in Table 4. The assessment of evidence quality using the GRADE approach is summarized in Table S2.

**TABLE 4:** GRADE.

Meta‐analysis		Risk of bias	Inconsistency^a^	Indirectness	Imprecision	Publication bias^b^	Quality of evidence
Static balance	tDCS	NS	NS	NS	S	NA	⊕⊕⊕○ Moderate
	SVT	S	S	NS	NS	NA	⊕⊕○○ Low
Dynamic balance	tDCS	NS	NS	NS	NS	NA	⊕⊕⊕⊕ High
	SVT	NS	S	NS	S	NA	⊕⊕○○ Low

Abbreviations: NA, not applicable; NS, not serious; S, serious; SVT, stroboscopic vision training; tDCS, transcranial direct current stimulation.

^a^ Substantial heterogeneity.

^b^ Not assessable due to small sample size.

## 4 Discussion

In this review, the effects of tDCS or SVT combined with balance training were evaluated on postural control performance in individuals with CAI. The results of the meta‐analysis showed significant differences in the effectiveness of these approaches on static or dynamic balance, which emphasizes the need to select evidence‐based protocols and further investigate synergistic effects.

### 4.1 Effect of tDCS Training on the Balance Performance

Our review identified five eligible studies on transcranial electrical stimulation for balance enhancement in CAI. Overall, the pooled analysis demonstrated small statistically significant improvements in both static and dynamic balance measures, characterized by low statistical heterogeneity that confirms consistent directionality across primary studies [27, 33, 38, 41]. In line with the primary studies, the meta‐analytic findings confirm that tDCS enhances postural control primarily when integrated into structured exercise programs rather than as a standalone intervention. These improvements are neurophysiologically driven by heightened synaptic plasticity and enhanced postural anticipatory adjustments, which increase recruitment of ankle‐stabilizing musculature (such as the tibialis anterior and peroneus longus) and improve joint position sense [42]. However, it appears that the effectiveness of tDCS is strongly influenced by the stimulation target within the brain.

Two studies that focused on the supplementary motor area (SMA) [26, 27] produced consistent results both observed that balance training alone significantly improved neural and functional measures, whereas adding active tDCS provided no additional benefit compared with sham stimulation. This pattern, where training is effective but tDCS adds no measurable advantage, mirrors findings from other populations. For example, a study in young healthy adults reported that cerebellar tDCS did not enhance learning of a complex whole‐body balance task [43]. That outcome was attributed to “ceiling effects,” meaning that performance had already reached a near‐optimal level through training, leaving minimal room for further improvement. Under such conditions, additional stimulation such as tDCS may fail to induce further neural adaptation because plasticity in the targeted circuit has already reached saturation [43]. Therefore, current evidence suggests that in SMA‐based protocols, training alone primarily drives improvement, and no additional contribution of tDCS can be clearly identified.

Conversely, two recent investigations [28, 39] targeting the primary motor cortex (M1) which governs the execution of motor commands to ankle‐stabilizing musculature revealed more nuanced and sometimes contradictory findings, emphasizing the influence of methodological details. Ge et al. [28] applied HD‐tDCS over M1 in combination with dynamic task‐relevant equilibrium training using a Bosu ball and reported marked synergistic enhancements. This indicates that simultaneous M1 stimulation during challenging balance tasks may strengthen neural processing and motor coordination. Conversely, Zhang et al. [39], who also targeted M1 but employed a different protocol, found that while tDCS alone improved static balance, training remained essential for dynamic balance improvements, and combining tDCS with training produced no superiority over sham conditions. Discrepancies between these trials can be explained by differences in task practice, stimulus parameters, and baseline deficit severity. When evaluating these outcomes in light of methodological quality, the RoB 2 and GRADE certainty of evidence must be carefully considered. Although tDCS combined with balance training yielded consistent positive effects with low statistical heterogeneity, the overall quality of evidence was rated as moderate due to inherent limitations in primary trials, particularly regarding allocation concealment, blinding of outcome assessors, and potential risk of bias in subjective functional domains. These methodological nuances temper the strength of clinical recommendations regarding M1 modulation [28, 39]. Such discrepancies can be explained by considering the distinct functional contributions of the stimulated brain regions, the mechanisms of tDCS, their interactions with task practice, and differences in patient populations. In SMA‐focused interventions, the targeted area is primarily associated with high‐level cognitive‐motor operations such as movement planning and initiation. However, since not all individuals with CAI exhibit balance deficits, the potential to detect improvement may be limited if most participants lack such impairments at baseline [41]. Theoretically, anodal tDCS could improve the efficiency of neural circuits involved in motor preparation [44]. Yet, functional impairments in CAI often arise at stages of automatic execution, rapid feedback control, and reflex‐like postural corrections processes governed mainly by the primary motor cortex, cerebellum, and subcortical pathways [42]. Consequently, modulating excitability within the SMA, either alone or combined with general training, appears insufficient to elicit functional changes that transfer to dynamic postural control in this population.

In contrast, the dual‐component approach used by Ge et al. [28] hinges on two main principles. First, enhancing cortical excitability via anodal tDCS to the motor cortex, where prior electrophysiological data show elevated pyramidal cell activity in M1 [45] and increased motor evoked potential amplitude in leg muscles [46]. Second, and more importantly, synchronizing this elevated cortical state with the timing of the training task is a well‐established factor in promoting effective neuromodulation [28]. Essentially, tDCS “primes” the brain for learning, whereas the concurrent training teaches the system the desired motor patterns [47]. This synergy enhances sensorimotor integration. During training, proprioceptive inputs from the ankle are processed by a more excitable and responsive motor cortex, allowing faster and more accurate interpretation of sensory data and more coordinated muscle responses [48, 49]. This mechanism is particularly vital for mediolateral stability control, which requires quick and precise activation of peroneal muscles to prevent ankle inversion trauma [50, 51]. Therefore, the moderate yet meaningful effect observed by Ge et al. can be attributed to the optimal alignment between stimulation target, timing, and task demands.

### 4.2 Effect of SVT on the Balance Ability

Results from the five studies examining balance training with stroboscopic glasses suggest that this method can serve as an effective complementary intervention.

SVT activates the sensory reweighting mechanism by creating controlled and intermittent disruption of visual inputs and reduces over‐reliance on vision. SVT by creating intermittent visual conditions reduces dependence on visual input and increases reliance on proprioceptive and vestibular information [52]. This approach is particularly beneficial in individuals with CAI, who frequently present with proprioceptive deficits, resulting in statistically significant enhancements in dynamic balance, whereas static balance improvements were less pronounced. Furthermore, a comparative synthesis indicates that our pooled meta‐analytic effect sizes aligned closely with the primary findings reported in the original trials, confirming a robust enhancement in dynamic stability. However, minor variations in effect magnitudes across individual studies were primarily driven by differences in training duration and baseline clinical instability levels. This discrepancy may be attributed to the relatively low strobe level (level 3; 100/150 ms) used during training, which might not have been sufficient to challenge static balance tasks. In contrast, dynamic tasks, being inherently more demanding, appear to benefit from this level of visual perturbation. Studies such as Uzlaşır et al. and Lee et al. [20, 40] primarily demonstrated improvements in functional and dynamic balance. In these studies, the main proposed mechanism is sensory reweighting [12].

Other studies investigated neurophysiological changes induced by SVT. Uzlaşır et al. [21] observed increased theta and alpha activity in the motor cortex, indicating heightened neural focus and processing during visual disruption. Choi et al. [16] also reported improved joint position sense accuracy, suggesting enhanced proprioceptive acuity. Such findings point to neural reorganization plastic changes in cortical function that strengthen connections between sensory and motor regions [21, 53]. Visual limitation appears to intensify proprioceptive processing within sensory cortices and reinforce efficient sensorimotor communication, ultimately improving balance control through enhanced non‐visual feedback.

The study by Kim et al. [23] effectively bridges these findings. The authors reported moderate improvements in perceived stability and very large improvements in dynamic balance. This pattern suggests that subjective and objective improvements likely originate from a shared central mechanism retraining the nervous system to utilize non‐visual inputs more effectively. Stroboscopic conditions compel participants to depend on proprioceptive and vestibular signals. Repeated successful balance experiences under these conditions facilitate sustained perceptual‐neural adaptations, including improved visual attention and sensitivity (i.e., lower central and peripheral motion coherence thresholds) [31], stronger coupling between cortical oscillations and postural control [54], and optimized muscle activation patterns during dynamic tasks [55]. As a result, the CNS develops greater confidence in non‐visual sensory feedback, yielding measurable gains in both subjective stability scores and objective performance tests. This adaptation reflects functional optimization rather than mere perceptual change the brain can now process proprioceptive information more efficiently and initiate muscular responses with shorter latency and higher precision. Overall, evidence indicates that SVT induces changes at both neurosensory and functional levels [21]. However, it should be noted that the pooled effect sizes for SVT were accompanied by substantial heterogeneity (*I* ^2^ = 93.22% for static balance and 89.23% for dynamic balance). This high heterogeneity may be attributed to differences in intervention protocols (e.g., strobe level, training duration, and frequency), variations in outcome measures, and differences in participant characteristics (e.g., severity of CAI and baseline balance ability). Therefore, the pooled effect sizes for SVT should be interpreted with caution. The intervention presents a controlled challenge by creating a temporary safe visual deficit. According to theoretical models, the CNS compensates through two complementary mechanisms: immediate sensory reweighting within subcortical networks and gradual cortical reorganization via neuroplasticity [40]. The combined outcome of these processes is enhanced dynamic balance, improved proprioceptive acuity, and increased subjective stability, establishing SVT as a multidimensional and effective rehabilitation tool. A theoretical tension should be acknowledged: although SVT is proposed to enhance sensory reweighting by disrupting visual input, proprioception is itself often impaired in individuals with CAI due to damage to mechanoreceptors and altered afferent feedback. This raises the question of what reliable input the CNS actually reweights toward when vision is disrupted. One possibility is that SVT may enhance central processing of remaining proprioceptive and vestibular signals, even if they are suboptimal, or that the benefits arise from improved use of vestibular inputs or cognitive strategies. The pooled effect sizes for SVT were accompanied by substantial heterogeneity and lower GRADE certainty ratings, driven primarily by imprecision (small sample sizes) and the impossibility of participant/therapist blinding inherent to visual perturbation protocols (high risk of performance and detection bias under RoB 2). Consequently, although SVT exhibits large point estimates for dynamic postural control, the lower quality of evidence cautions against over‐interpreting these gains as definitive without further rigorous high‐power trials.

### 4.3 Clinical Implications

tDCS is a noninvasive intervention that modulates motor cortex excitability and, when applied concurrently with motor tasks such as balance exercises, can promote activity‐dependent neuroplasticity. Current evidence in individuals with chronic ankle instability (CAI) suggests that anodal tDCS over the lower extremity motor cortex (Cz) may improve static balance, whereas its effects on dynamic balance appear only when combined with balance training. In contrast, stimulation of the SMA has not demonstrated additional benefits over sham. However, due to the limited number of studies and heterogeneity in protocols and outcome measures, firm conclusions cannot be drawn. In clinical practice, the choice between tDCS and SVT should be guided by patient characteristics, available resources, and specific treatment goals. For individuals who may benefit from enhanced cortical excitability and more consistent evidence, tDCS may be considered. For those who may respond to sensory manipulation and have access to stroboscopic glasses, SVT may be appropriate, particularly for improving dynamic balance. However, both interventions demonstrated their greatest added value when combined with balance training, and neither should be used as a stand‐alone treatment. The evidence base is currently insufficient to recommend one approach over the other definitively. SVT is portable, practical, and easily integrated into return‐to‐sport programs. The effectiveness of SVT appears to be influenced by the choice of strobe frequency. Although a fixed level of 3 (100/150 ms) has been commonly used and considered safe, evidence suggests that a progressive individualized approach where the strobe level is increased based on the patient's performance may yield superior outcomes. This method ensures an optimal and continuous challenge to the sensorimotor system, promoting greater adaptations in dynamic postural control. Although the training protocols varied across the included studies, a key insight emerged from the comparison between the two interventions. In tDCS studies, the stimulation site appeared more critical to outcomes than the specific type of balance exercise performed. In contrast, for SVT, the nature of the training task and the progression of the strobe level rather than the visual manipulation alone were the primary determinants of successful outcomes. Further high‐quality studies with standardized protocols are needed to establish definitive clinical guidelines. Across both modalities, clinical success is governed by the risk of bias in primary studies and the strength of the evidence base. Practitioners must recognize that neither tDCS nor SVT should serve as standalone modalities; their primary value lies in acting as neuroplastic adjuvants to structured progressive balance exercise programs.

### 4.4 Future Research and Limitations

This systematic review has two main categories of limitations: those arising from the included primary studies and those related to the review process itself.

#### 4.4.1 Limitations of the Included Studies

A key limitation is the difficulty of proper blinding in studies using stroboscopic glasses. Due to the nature of these interventions, neither participants nor therapists could be completely unaware of the treatment, which introduces a risk of performance and detection bias. This limitation may have led to an overestimation of intervention effects through contextual or placebo influences. Selection bias was also a concern in some studies, as random allocation and concealment procedures were not always clearly reported. Additionally, several studies had small sample sizes, which reduced statistical power and made the estimates less reliable. Furthermore, considerable variation in training protocols, including differences in type, intensity, and duration, contributed to heterogeneity and limited our ability to compare findings across studies and draw firm overall conclusions. Moreover, none of the included studies reported adequate allocation concealment (Item 6 of the PEDro scale), which may have introduced selection bias.

#### 4.4.2 Limitations of the Review Process

The search was restricted to eight major databases and only English‐language papers, which may have resulted in the omission of relevant studies published in other databases or languages. To minimize this limitation, we performed a manual search of the reference lists of included studies and relevant reviews. Moreover, we were unable to obtain raw or additional data from some authors, which prevented deeper subgroup or sensitivity analyses. The observed heterogeneity could not be statistically accounted for by gender distribution, intervention frequency, or the total number of training sessions, as the available data did not permit such analyses. Future research with standardized and transparent reporting of these characteristics is needed to enhance the robustness and clarity of the evidence. Finally, the small number of high‐quality studies with consistent designs limited the strength of the quantitative synthesis.

#### 4.4.3 Future Research Directions

Future studies should adhere to standardized and transparent reporting of intervention protocols and participant characteristics to facilitate more robust comparisons and meta‐analyses. Larger well‐designed randomized controlled trials with appropriate blinding strategies and longer follow‐up periods are needed to confirm the findings of this review. Future studies should also prioritize proper allocation concealment (Item 6 of the PEDro scale) to enhance methodological rigor and reduce the risk of selection bias.

## 5 Conclusion

This systematic review and meta‐analysis compared the effects of balance training combined with either tDCS or SVT on postural control in individuals with CAI. The findings reveal that while both interventions are promising adjuncts, the strength and consistency of their evidence differ considerably. tDCS provides small but reproducible improvements, supported by consistent findings across studies, making it preferable when treatment reliability and predictability are primary concerns. In contrast, SVT yields larger improvements, particularly in dynamic balance, yet these effects are highly variable and based on less certain evidence, which limits their generalizability to clinical practice. Consequently, SVT may be considered for individuals who tolerate sensory perturbation well and can adhere to structured, progressive protocols, but its application should be accompanied by careful monitoring. Neither intervention should replace conventional balance training; rather, both should serve as complementary tools selected on the basis of individual patient characteristics, including baseline sensory reliance, cognitive status, and access to equipment. The absence of head‐to‐head trials directly comparing tDCS and SVT remains a critical gap in the literature. Future research should prioritize active‐comparator randomized controlled trials to establish whether the large effects observed for SVT are reproducible under standardized conditions, as well as dose‐response studies to determine optimal stimulation parameters and stroboscopic levels for different patient subgroups. Until such evidence becomes available, clinical decisions must remain individualized, and clinicians should weigh the trade‐off between effect magnitude and evidence robustness when choosing between these two approaches.

## Author Contributions

**Ali Shamsi Majelan:** conceptualization, data curation, funding acquisition, investigation, methodology, project administration, resources, software, supervision, validation, visualization. **Omid Shahani:** conceptualization, data curation, formal analysis, funding acquisition, investigation, methodology, project administration, resources, software, visualization, writing – original draft preparation, writing – review and editing. **Atekeh Keivani:** conceptualization, data curation, formal analysis, software, writing – original draft preparation, writing – review and editing. **Mahla Armanfar:** conceptualization, data curation, formal analysis, writing – original draft preparation, writing – review and editing. **Brice Picot:** conceptualization, data curation, formal analysis, investigation, methodology, project administration, resources, writing – original draft preparation.

## Funding

The authors have nothing to report.

## Ethics Statement

The authors have nothing to report.

## Consent

The authors have nothing to report.

## Conflicts of Interest

The authors declare no conflicts of interest.

## Permission to Reproduce Material From Other Sources

The authors have nothing to report.

## Supporting information


Supporting Information S1

**Table S1:** Search Strategy.

**Table S2:** GRADE summary of findings.

## Data Availability

Data are available from the corresponding author upon reasonable request.
