# Supplementary material for: Effects of Balance Training Combined With Either Transcranial Direct Current Stimulation or Stroboscopic Vision Training on Balance Performance in Individuals With Chronic Ankle Instability: A Systematic Review and Meta‐Analysis
Source: J Foot Ankle Res. 2026 Sep 19;19(3):e70210. doi: 10.1002/jfa2.70210 (PMC13589631; doi:10.1002/jfa2.70210)
Supplement: Supplementary file 1 [file JFA2-19-e70210-s001.docx]

Table S3. GRADE summary of findings

| Type of intervention | | Absolute  effect* | No. of studies | Effect estimate (Hedges' g, 95% CI) | Certainty  of evidence | Explanation |
| --- | --- | --- | --- | --- | --- | --- |
| tDCS | Dynamic balance | - | 3 RCTs | 0.252 (−0.106,0.610) | High | Not downgraded |
|  | Static balance | - | 4 RCTs | 0.303 (0.143, 0.463) | Moderate | Downgraded by one levels because of serious imprecision. |
| SVT | Dynamic balance | - | 3 RCTs | 1.086 (0.179, 1.993) | Low | Downgraded by two levels because of serious inconsistency and serious imprecision. |
|  | Static balance | - | 3 RCTs | 2.131 (0.718, 3.544) | Low | Downgraded by two levels because of serious inconsistency and publication bias. |

*Because continuous outcomes were synthesized using standardized mean differences (Hedges' g), absolute effect estimates were not applicable and are therefore not reported.SVT: Stroboscopic Vision Training, tDCS: Transcranial Direct Current Stimulation, RCTs: Randomized Clinical Trail study.
