# Supplementary material for: Effects of Balance Training Combined With Either Transcranial Direct Current Stimulation or Stroboscopic Vision Training on Balance Performance in Individuals With Chronic Ankle Instability: A Systematic Review and Meta‐Analysis
Source: J Foot Ankle Res. 2026 Sep 19;19(3):e70210. doi: 10.1002/jfa2.70210 (PMC13589631; doi:10.1002/jfa2.70210)
Supplement: Supplementary file 2 [file JFA2-19-e70210-s002.docx]

**Sensitivity Analysis**

Sensitivity analysis, conducted using correlation coefficients (r) of 0.25 and 0.75, demonstrated that the findings remained robust, with no statistically significant changes compared to the primary analysis (r=0.50). These results were consistent across both static and dynamic balance assessments. Further details regarding these sensitivity analyses are illustrated in Figures 1 through 4.

**Static Balance**

**Sensitivity analysis with r=0.25**

Overall: I^2^=87.49%, p=0.0001

tDCS: I^2^=0.0%, p=0.730

SVT: I^2^=88.07%, p=0.0001

**^Fig 1.^** ^Sensitivity analysis static balance (r=0.25)^

**Sensitivity analysis with r=0.75**

Overall: I^2^=86.70%, p=0.0001

tDCS: I^2^=0.0%, p=0.735

SVT: I^2^=88.06%, p=0.0001

**^Fig 2.^** ^Sensitivity analysis static balance (r=0.75)^

**Dynamic Balance**

**Sensitivity analysis with r=0.25**

Overall: I^2^=83.74%, p=0.0001

tDCS: I^2^=0.0%, p=0.624

SVT: I^2^=88.36%, p=0.0001

**^Fig 3.^** ^Sensitivity analysis dynamic balance (r=0.25)^

**Sensitivity analysis with r=0.75**

Overall: I^2^=73.19%, p=0.002

tDCS: I^2^=0.0%, p=0.753

SVT: I^2^=80.02%, p=0.007

**^Fig 4.^** ^Sensitivity analysis dynamic balance (r=0.75)^
