# Supplementary material for: Effects of Balance Training Combined With Either Transcranial Direct Current Stimulation or Stroboscopic Vision Training on Balance Performance in Individuals With Chronic Ankle Instability: A Systematic Review and Meta‐Analysis
Source: J Foot Ankle Res. 2026 Sep 19;19(3):e70210. doi: 10.1002/jfa2.70210 (PMC13589631; doi:10.1002/jfa2.70210)
Supplement: Supplementary file 3 [file JFA2-19-e70210-s003.docx]

Table S1. Search strategy

| Studies published in English from inception to June 20, 2026 | | |
| --- | --- | --- |
| Database | Search | Result |
| Web of Sciences | “Transcranial Direct Current Stimulation” OR “TDCs” OR “Electrical Stimulations” OR “Electroshock” OR “Non-invasive brain stimulation” OR “Cathodal Stimulation” OR “Excitability” (All Fields) and “Chronic ankle instability” OR “CAI” OR “Ankle sprain” OR “ankle injuries” OR “functional ankle instability” OR “Lateral ankle sprain” (All Fields) and “Balance” OR “Postural control” OR “COP” OR “Dynamic” OR “Static” OR “Postural sway” OR “Stability” OR “Time to stability” (All Fields) | 102 |
|  | “Stroboscopic” OR “Strobe glasses” OR “Stroboscopic vision” OR “motion perception” OR “Motion tracking” OR “Visual skills” (All Fields) and “Chronic ankle instability” OR “CAI” OR “Ankle sprain” OR “ankle injuries” OR “functional ankle instability” OR “Lateral ankle sprain” (All Fields) and “Balance” OR “Postural control” OR “COP” OR “Dynamic” OR “Static” OR “Postural sway” OR “Stability” OR “Time to stability” (All Fields) | 41 |
| Scopus | (TITLE-ABS-KEY ("Transcranial Direct Current Stimulation" OR "TDCs" OR "Electrical Stimulations" OR "Electroshock" OR "Non-invasive brain stimulation" OR "Cathodal Stimulation" OR "Excitability") AND TITLE-ABS-KEY ("Chronic ankle instability" OR "CAI" OR "Ankle sprain" OR "ankle injuries" OR "functional ankle instability" OR "Lateral ankle sprain") AND TITLE-ABS-KEY ("Balance" OR "Postural control" OR "COP" OR "Dynamic" OR "Static" OR "Postural sway" OR "Stability" OR "Time to stability")) | 75 |
|  | ( TITLE-ABS-KEY ( "Stroboscopic" OR "Strobe glasses" OR "Stroboscopic vision" OR "motion perception" OR "Motion tracking" OR "Visual skills" ) AND TITLE-ABS-KEY ( "Chronic ankle instability" OR "CAI" OR "Ankle sprain" OR "ankle injuries" OR "functional ankle instability" OR "Lateral ankle sprain" ) AND TITLE-ABS-KEY ( "Balance" OR "Postural control" OR "COP" OR "Dynamic" OR "Static" OR "Postural sway" OR "Stability" OR "Time to stability" ) ) | 26 |
| Science direct | “Transcranial Direct Current Stimulation” AND “Stroboscopic” AND “Chronic ankle instability” OR "CAI" AND “Balance” | 189 |
| PubMed | ("Transcranial Direct Current Stimulation"[All Fields] OR "TDCs"[All Fields] OR "Electrical Stimulations"[All Fields] OR "Electroshock"[All Fields] OR "Non-invasive brain stimulation"[All Fields] OR "Cathodal Stimulation"[All Fields] OR "Excitability"[All Fields]) AND ("Chronic ankle instability"[All Fields] OR "CAI"[All Fields] OR "Ankle sprain"[All Fields] OR "ankle injuries"[All Fields] OR "functional ankle instability"[All Fields] OR "Lateral ankle sprain"[All Fields]) AND ("Balance"[All Fields] OR "Postural control"[All Fields] OR "COP"[All Fields] OR "Dynamic"[All Fields] OR "Static"[All Fields] OR "Postural sway"[All Fields] OR "Stability"[All Fields] OR "Time to stability"[All Fields]) | 63 |
|  | ("Stroboscopic"[All Fields] OR "Strobe glasses"[All Fields] OR "Stroboscopic vision"[All Fields] OR "motion perception"[All Fields] OR "Motion tracking"[All Fields] OR "Visual skills"[All Fields]) AND ("Chronic ankle instability"[All Fields] OR "CAI"[All Fields] OR "Ankle sprain"[All Fields] OR "ankle injuries"[All Fields] OR "functional ankle instability"[All Fields] OR "Lateral ankle sprain"[All Fields]) AND ("Balance"[All Fields] OR "Postural control"[All Fields] OR "COP"[All Fields] OR "Dynamic"[All Fields] OR "Static"[All Fields] OR "Postural sway"[All Fields] OR "Stability"[All Fields] OR "Time to stability"[All Fields]) | 25 |
| EBSCO | ("Transcranial Direct Current Stimulation"[All Fields] OR "TDCs"[All Fields] OR "Electrical Stimulations"[All Fields] OR "Electroshock"[All Fields] OR "Non-invasive brain stimulation"[All Fields] OR "Cathodal Stimulation"[All Fields] OR "Excitability"[All Fields]) AND ("Chronic ankle instability"[All Fields] OR "CAI"[All Fields] OR "Ankle sprain"[All Fields] OR "ankle injuries"[All Fields] OR "functional ankle instability"[All Fields] OR "Lateral ankle sprain"[All Fields]) AND ("Balance"[All Fields] OR "Postural control"[All Fields] OR "COP"[All Fields] OR "Dynamic"[All Fields] OR "Static"[All Fields] OR "Postural sway"[All Fields] OR "Stability"[All Fields] OR "Time to stability"[All Fields]) | 98 |
|  | ("Stroboscopic"[All Fields] OR "Strobe glasses"[All Fields] OR "Stroboscopic vision"[All Fields] OR "motion perception"[All Fields] OR "Motion tracking"[All Fields] OR "Visual skills"[All Fields]) AND ("Chronic ankle instability"[All Fields] OR "CAI"[All Fields] OR "Ankle sprain"[All Fields] OR "ankle injuries"[All Fields] OR "functional ankle instability"[All Fields] OR "Lateral ankle sprain"[All Fields]) AND ("Balance"[All Fields] OR "Postural control"[All Fields] OR "COP"[All Fields] OR "Dynamic"[All Fields] OR "Static"[All Fields] OR "Postural sway"[All Fields] OR "Stability"[All Fields] OR "Time to stability"[All Fields]) | 44 |
| Embase | ('transcranial direct current stimulation':ti,ab,kw OR 'tdcs':ti,ab,kw OR 'electrical stimulations':ti,ab,kw OR 'electroshock':ti,ab,kw OR 'non-invasive brain stimulation':ti,ab,kw OR 'cathodal stimulation':ti,ab,kw OR 'excitability':ti,ab,kw) AND ('chronic ankle instability':ti,ab,kw OR 'cai':ti,ab,kw OR 'ankle sprain':ti,ab,kw OR 'ankle injuries':ti,ab,kw OR 'functional ankle instability':ti,ab,kw OR 'lateral ankle sprain':ti,ab,kw) AND ('balance':ti,ab,kw OR 'postural control':ti,ab,kw OR 'cop':ti,ab,kw OR 'dynamic':ti,ab,kw OR 'static':ti,ab,kw OR 'postural sway':ti,ab,kw OR 'stability':ti,ab,kw OR 'time to stability':ti,ab,kw) | 53 |
|  | ('stroboscopic':ti,ab,kw OR 'strobe glasses':ti,ab,kw OR 'stroboscopic vision':ti,ab,kw OR 'motion perception':ti,ab,kw OR 'motion tracking':ti,ab,kw OR 'visual skills':ti,ab,kw) AND ('chronic ankle instability':ti,ab,kw OR 'cai':ti,ab,kw OR 'ankle sprain':ti,ab,kw OR 'ankle injuries':ti,ab,kw OR 'functional ankle instability':ti,ab,kw OR 'lateral ankle sprain':ti,ab,kw) AND ('balance':ti,ab,kw OR 'postural control':ti,ab,kw OR 'cop':ti,ab,kw OR 'dynamic':ti,ab,kw OR 'static':ti,ab,kw OR 'postural sway':ti,ab,kw OR 'stability':ti,ab,kw OR 'time to stability':ti,ab,kw) | 21 |
| Cochrane Library | “Transcranial Direct Current Stimulation” OR “TDCs” OR “Electrical Stimulations” OR “Electroshock” OR “Non-invasive brain stimulation” OR “Cathodal Stimulation” OR “Excitability” in Title Abstract Keyword AND “Chronic ankle instability” OR “CAI” OR “Ankle sprain” OR “ankle injuries” OR “functional ankle instability” OR “Lateral ankle sprain” in Title Abstract Keyword AND “Balance” OR “Postural control” OR “COP” OR “Dynamic” OR “Static” OR “Postural sway” OR “Stability” OR “Time to stability” in Title Abstract Keyword AND “Stroboscopic” OR “Strobe glasses” OR “Stroboscopic vision” OR “motion perception” OR “Motion tracking” OR “Visual skills” in Title Abstract Keyword - (Word variations have been searched) | 18 |
| Google Scholar | (“Transcranial Direct Current Stimulation” OR “TDCs” OR “Electrical Stimulations” OR “Electroshock” OR “Non-invasive brain stimulation” OR “Cathodal Stimulation” OR “Excitability”) AND (“Chronic ankle instability” OR “CAI” OR “Ankle sprain” OR “ankle injuries” OR “functional ankle instability” OR “Lateral ankle sprain”) AND (“Balance” OR “Postural control” OR “COP” OR “Dynamic” OR “Static” OR “Postural sway” OR “Stability” OR “Time to stability”) | 15 |
|  | (“Stroboscopic” OR “Strobe glasses” OR “Stroboscopic vision” OR “motion perception” OR “Motion tracking” OR “Visual skills”) AND (“Chronic ankle instability” OR “CAI” OR “Ankle sprain” OR “ankle injuries” OR “functional ankle instability” OR “Lateral ankle sprain”) AND (“Balance” OR “Postural control” OR “COP” OR “Dynamic” OR “Static” OR “Postural sway” OR “Stability” OR “Time to stability”) | 6 |
